# Editorial: The Evolution of Music

**DOI:** 10.3389/fpsyg.2020.595517

**Published:** 2020-10-28

**Authors:** Aleksey Nikolsky, Leonid Perlovsky

**Affiliations:** ^1^Braavo Enterprises, Los Angeles, CA, United States; ^2^Department of Psychology, Northeastern University, Boston, MA, United States

**Keywords:** interdisciplinary approach, scientific/humanitarian methods, ethnomusicology, empirical musicology, musilanguage, musicological analysis, organology, geomusicology

Two decades ago, Wallin et al. (1999) opened a new page in the quest for the origins of music by integrating the methodologies and data coming from numerous disciplines:

- physical anthropology
- paleoneurobiology
- ethology
- biocultural evolution
- systematic musicology
- semiotics
- historical linguistics
- developmental psychology

This volume updates this multi-disciplinary approach by further advancing the fields defined by Wallin et al. and by introducing and specifying new fields of inquiry:

- organology
- musicological analysis
- geomusicology
- demography
- information theory
- statistic modeling
- (paleo)aesthetics
- (paleo)phonology
- biological motion

In addition, this volume addresses a number of interdisciplinary problems that were identified by the contributing authors^1^. The latter issue has recently become critical: the very idea of multidisciplinary study of music has been questioned. There is a growing conviction amongst Western scholars of ethnomusicological background that humanities and sciences are fundamentally split, and the scientific approach somehow introduces an “anti-humanitarian” bias (Parncutt, 2017). According to this view, specialists in sciences should adjust their methodologies to comply with the conventions of political correctness currently adopted by many Western specialists in musicology and social sciences. This view exploits the argument that the public trust in science supports the “scientific hegemony” in an ongoing cultural “warfare” between disciplines of art and science, thereby precluding the advance in human knowledge (Cohen, 2001). This argument was introduced half-a-century ago in a popular book by Snow (1964). Despite being convincingly debunked by Wilson (1998) and Gould (2003), it resurfaced again in the ideology of “new mysterianism,” propagated by Chomsky (2016) and McGinn (2015). Their intellectual weight has made the call for “humanizing” science more appealing to scientists.

Mysterianist and anti-scientific sentiments found fertile ground amongst many musicians and musicologists that regard a scientific approach as being “bound to the science lab” and therefore irrelevant to music practice—even detrimental to the expressive efficacy of music-makers (Woody, 2004). Indeed, differences in jargon between musicological^2^ and psychoacoustic disciplines set a barrier against co-understanding, which preserves the popular myths about perception/production of music (Juslin et al., 2012). Many believe that music is incommensurable, irrational, and mystic—unsuitable for objective investigation (Dubal, 1985) and demanding an intuitive approach (Woody, 2000).

A big role in the spread of this attitude has been played by the ongoing trend of “scientism” in influential Western schools of composition, and the critical acclaim they have been receiving from well-established musical critics and musicologists (Regelski, 2014). Overwhelmingly, the music works composed after WWII and esteemed by academia bear a strong flavor of scholasticism which resembles that of religious scholarly traditions of pre-industrial cultures. The New Music of the West has deliberately exploited the “scientific” image in employing an invention-like approach, equating the method of strictly following a set of newly invented abstract compositional principles to mathematics (Babbitt, 2003). Such music usually violates the psycho-physiological restraints (Thomson, 2010), contributing to the public impression of its fundamental indigestibility (Lerdahl, 1992).

However, in toto, the situation is far from a global scientific/humanitarian schism: science does not defy humanities—it only corrects erroneous traditional beliefs. The only humanitarian discipline that consistently defies scientific methodology is Western ethnomusicology. In the past 40 years it has adopted the view that there is no such thing as “music” but myriad of “musics” (Becker, 1986), each requiring its unique frame of investigation (Nettl, 2010). The very application of a scientific method is viewed here as exercising Eurocentric political power over non-European cultures (Messner, 1993)—seen even in merely calling a non-European artifact “music” (Bohlman, 1999). This highly politicized philosophy resulted in abandoning comparative studies of music systems (Savage and Brown, 2013), of the origins and evolution of music (Nettl, 2005), and of music analysis (Nattiez, 2012), especially pronounced in the US and UK ethnomusicological schools (Zemtsovsky, 2002).

General shift of Western ethnomusicology away from comparative musicology to fractured sociomusicology of isolated musical communities was inspired by concerns for compensating for the earlier Eurocentric bias in ethnographic research prior to the mid-twentieth century. Late twentieth century ethnomusicologists avoid direct comparisons between different music cultures altogether, especially those involved in establishing cultural evolution (Nettl, 2010, p. 70–92). This revisionism relies on a systemic over-evaluation of the scope and limitations of purely emic approach to the study of music, in combination with a drastic under-evaluation of the advantages of the etic approach (see Nikolsky et al.).

The special issue of “*The World of Music,”* the journal of the International Music Council, (Vol. 22, No. 3, 1980), was dedicated to the discussion of the possibility to draw a general history of music, explore its origins, and formulate generalities in evolution of different musical cultures. Its overwhelming negative conclusions reflected the emerging trend of replacing the comparative studies that had been conducted within the field of systematic musicology by the disconnected studies of each musical culture, presumably entitled to its own unique “history” (Myers, 1993). Accordingly, the official name of the discipline was changed from “systematic musicology” to “ethnomusicology.” Noteworthy is the radical inversion of the views on comparative studies by the “old-timers,” such as Bruno Nettl, from positive in 1968 (Nettl and Blum, 1968) to negative in 2005 (Nettl, 2005).

Responding to Gourlay's call for ethnomusicology to abandon the “pretense of objectivity” in favor of “humanization” (Gourlay, 1982), Western ethnomusicologists started viewing their mission as “the study of people making music” rather than “the study of music” as the term “musicology” suggests (Titon, 2015). They substituted the study of text (that has traditionally been considered the “primary reality” for studying the arts) with the study of people's behaviors, causing a methodologic shift away from the analysis of music to sociology (Zemtsovsky, 1997)—exemplified by the following quotation: “Unless the formal analysis begins as an analysis of the social situation that generates the music, it is meaningless” (Blacking, 1974, p. 71). Analysis *per se* has acquired the reputation of a form of “composing” the analyzed piece of music on the part of an analyst, thereby distorting the original meaning of that music (Agawu, 2004). In effect, such a position deprived Western ethnomusicology of an empirical and objective foundation.

This trajectory is polarly opposite to the trend of equipping musicology with means of scientific research, prevalent in the former Soviet Bloc countries (Myers, 1993)^3^. Integration of scientific and humanitarian knowledge has been a long tradition there since Lobachevsky^4^ (Grigoryan, 2011)—affecting scholarship of all orientations: materialistic (Aleksei Losev), neo-Christian (Pavel Florensky), and esoteric (Peter Ouspensky). Music-related studies made no exception: noteworthy were such figures as Losev^5^, Maykapar^6^, and Samoilov ^7^. All major Russian researchers of psychoacoustics could interpret/perform music at a professional level or/and compose or arrange music: Sofia Beliayeva-Ekzempliarskaya, Nikolai Garbuzov, Boris Teplov, Aleksey Ogolevets, Alexander Volodin, Yevgenii Nazaikinsky, and Yuri Rags. In 1944, Institute of History of Arts at the USSR Academy of Sciences was founded for scientific investigation of the arts (including music).

In the West too, there are ongoing attempts to bridge humanities and science by making musicology “empirical” (Clifton, 1983; Deutsch, 1996; Gjerdingen, 1999; Clarke and Cook, 2004; Honing, 2006; Baily, 2009; Schneider and Ruschkowski, 2011; Kendall and Lipscomb, 2013).

After all, every musician routinely conducts informal experiments: performers evaluate alternative ways of rendering music; studio-musicians experiment with various arrangements, and ear-trainers test students. Experimental trial of the premises of musical theory was discussed at the year-long seminar at Stanford, involving representatives of humanitarian and scientific disciplines (e.g., Lerdahl, Narmour, Gjerdingen, Bharucha, Palmer, and Krumhansl)—with the outcomes published in a special issue (1996) of “Music Perception.” Since then, this idea has attracted attention of many scholars..

This is the direction we pursue in this collection of papers. It starts with Harvey overviewing the origins of music and presenting a theory of music that reflects a “society of selves” through social cooperation. Harvey attributes the invention of music to the promotion of group coherence and personal well-being.

Montagu informs readers without music education about the capacities of early humans, their possible musical behavior, and the overall evolution of musicality. Special attention is given to the development of musical instruments, which provides a window to the reconstruction of the musicking practices of the past.

Malloch and Trevarthen present an account of music in terms of human cognition and biology, with emphasis on musical education. They show how cultural life and learning depend on the motivation for sharing projects of thought and action, musically. Music empowers the transmission of the narratives of one's “inner life” in bodily movements. This ongoing practice must have transformed the primate brain for the affective regulation of social learning, thereby determining the evolution of human musical mind.

Brown updates his widely acknowledged musilanguage theory (Brown, 2000) by proposing the joint prosodic origin of proto-language and proto-music, where both shared specialization in emotional communication and neither featured scaled pitches. Brown introduces a “prosodic scaffold” model—i.e., specific vocal articulations and accompanying mimics/gestures forming signs for “acoustic pantomimes,” designed to express one's affective state. According to Brown, combinatorial and compositional mechanisms of utterances generated the affective prosody, designating characteristic patterns of global acoustic expression for common emotional states. This forged into national prosody that branched into proto-language and proto-music based on, respectively, dialogic and chorusing formats of communication—distinguished by different approach to timing. At this point, music acquired concise temporal organization and synchronicity of collective production/perception.

Nikolsky introduces the term “isophony” to refer to the tonal and rhythmic properties of the musilanguage system. This amendment, endorsed by Brown, corrects the mismatch between the psychoacoustic and musicological taxonomies of musical texture. Nikolsky formulates a set of clear structural distinctions between the most common textural types: heterophony, homophony, and polyphony—in comparison to “isophony.”

van der Schyff and Schiavio overview the key positions in evolutionary musicology, identifying the problems of the nature-or-culture antithesis. They elaborate on the “biocultural” approach exemplified in the work by Tomlinson (2015), to resolve these problems. The authors examine a range of supporting evidence for this approach vs. the “embodied” approach that regards music as a bio-cultural process governed by interaction of the enacted and social aspects. The authors cross-relate the current developments in evolutionary musicology, such as “enactivism” and “4E cognition,” and suggest how the biocultural and “enactivist” approaches can be improved.

Nikolsky brings together insights from semiotics, musicology, psychoacoustics, evolutionary biology, anthropology, ethology, linguistics, and geomusicology to coin a new line of inquiry revolving around the concept of expressive aspects of music—in contradistinction from phonetics and prosody in natural languages. Central in this approach is the role of tonal organization and the comparative analysis of the acoustic features of indigenous musical traditions and animal communication calls. Nikolsky offers a new method of multifactorial modal analysis of tonal organization and its graphic representation (“musogram”) and examines its pros and cons in light of the emic/etic antithesis. He argues that music evolved from animal communication through gradual substitution of “one-ended” communication with “two-ended,” where each of multiple aspects of expression acquired a repertory of proprietary signs for effective communication of emotional information, thereby remapping the animal-like instinctive correspondences between acoustic traits and affective states. The complete “semiotization” of all principal aspects of expression must have occurred no earlier than during the Neolithic “revolution” within the framework of the emerging bi-specific communication between humans and domestic animals—exemplified in the surviving pastoral culture of kulning.

Jan proposes yet another method of musicological analysis for tracking the lineage of diachronic evolution of specific musical structures based on the memetic approach to cultural changes. Elaborating on the research by Savage (2016), Jan combines the quantitative corpus-analysis techniques, adapted from molecular biology, with qualitative method of identifying perceptual-cognitive elements of music—“musemes”—revealed by music's motivic organization. This novel humanitarian-scientific integration promises a compelling and potentially testable means for studying the cultural evolution of music.

Lumaca et al. define a new interdisciplinary field of research—the contribution of neural constraints and biases on the cultural evolution of musical structures through the chain of cultural transmissions. Capacities of the human brain constrain acquisition, production, and reproduction of music. To illustrate this, the authors demonstrate a progressive diatonization of tonal organization in the multi-generational signaling game settings, which suggests that the smaller the information-processing bottleneck in individuals, the larger the pressures to regularize the music material. This poses new intriguing questions, such as the *r*ole of neural variability in music diversity—which is of greatest value for folk forms of music that entirely rely on oral transmission.

Podlipniak explores the Baldwinian evolutionary modeling in search for a compelling and testable theory for the phenomenon of human musicality, hypothesizing that it might constitute an adaptive phenomenon. In the Baldwin effect, animals, learn new behaviors that allow them to survive and reproduce in the changing environment. Podlipniak argues that the increasing group size of ancestral hominins required new mechanisms of “social consolidation” in the form of “collective imitation.” There is then a natural selection for the evolution of new circuitry for vocal learning to maintain social bonds, thereby reducing the cost of learning.

Nikolsky et al. describe and explain the “timbre-based music” as a special system of musicking, communication, psychological, and social usage, along with corresponding beliefs—placing it in the timeline of the evolution of music. Timbral music opposes conventional Western music by its personal orientation: musicking here occurs primarily for oneself and/or for close relatives/friends. Throughout northeastern Eurasia, “personal song” serves as an important means of individual identification and territorial marking, akin a passport, supporting individual's mental health under harsh environmental conditions. The authors use demographic, geomusicological, paleoenvironmental, organological, and paleophonological data to argue that Siberian timbre-oriented music is remnant of the pan-Eurasian prehistoric music tradition that originated from the Last Glacial Period. Western frequency-oriented music, with its reliance on collective production/perception, might have been exported from Africa (where the population was much denser) before the LGP.

Ravignani et al. draw a parallel between researching the evolutions of music and language. They call for a systemic revaluation of an empirical and scientific approaches as opposed to claims from Chomskyan and anthropological perspectives against the scientific study. Given the intellectual influence of Chomsky, this point is extremely important. It would benefit musicologists to follow the lead of phonologists in establishing the evolutionary chain of developments. Re-aligning musicological and linguistic methodologies can allow the developmental psychologists and ethologists to make better choices between musical and linguistic paradigms in their research.

Fenk-Oczlon reports an intriguing phenomenon: the number of vowels and pitch-classes in native languages and native music tend to match. This is most pronounced in cases of simple systems—tritonic musical vs. 3-vowel vocal systems—but also noticeable in very complex 12-element systems. The mean values also match at 5–7 elements. Such correspondence supports Brown's model by revealing the shared ground between musical and vowel pitches.

Ravignani and Madison analyze the phenomenon of isochrony across human music and speech against animal communication, integrating the data from mathematics, physics, signal processing, physiology, and neuroscience. They define the concept of isochrony and propose an evolutionary hypothesis to explain why amongst all animals it is only humans that possess superb isochronous perception which does not confer evolutionary advantage to modern humans.

Honing et al. use an EEG oddball paradigm to assess the neural sensitivity to isochronous or arrhythmic beats in two monkeys. This non-invasive EEG methodology enables a direct comparison of the perception of monkeys, non-human primates, and humans. The authors found the MMN responses to the isochronous pattern but no strong evidence for beat sensitivity, confirming the Gradual Audiomotor Evolution model (Merchant and Honing, 2014) that holds metric organization as a biological marker of human music.

Loui et al. raise an intriguing question of musical anhedonia through cross-examination of its rare case against a panel of neurotypical participants. Their findings demonstrate the categorically different decreased connectivity between auditory and reward systems, supporting the Mixed Origins of Music model (Altenmüller et al., 2013). The authors identify neural pathways engaged in music's operation as an affective signaling system.

Based on his extensive teaching experience and research, Crickmore asserts his earlier experimental study and the test settings for measuring “aesthetic emotions”—listeners' response to detected “musical emotions” expressed by music creators. His revised test paves the road for clarifying the relations between human emotions, genres, and personality.

Masataka demonstrates that young people with autism spectrum disorder display more interest to dissonant music than typically developed matched group. This indicates that neural diversity within autism spectrum might have played a role in the evolution of dissonant sad music—important for overcoming negative aesthetic emotions of cognitive dissonances (Masataka and Perlovsky, 2013).

Trulla et al. explain consonance of musical intervals based on “second order beats,”^8^ described by an approach borrowed from dynamical systems analysis—a quantitative index obtained from Recurrence Quantification Analysis. The novelty of this method is that it accounts for frequency ratio relationships plus temporal behavior. The authors confirm that musical consonance/dissonance has a mathematical foundation and that music perception, in general, and harmonic intervals, in particular, are a consequence of the entrainment of the nervous system with sound excitation.

Keller et al. provide a piece of evidence to support the Darwinian hypothesis of music's origin in sexual selection. In their study, a professional boys' choir was found to exhibit musical behavior essentially similar to male chorusing in many animal species. In female presence, bass singers instinctively emphasized and rose their singing formant—despite the conventional non-acceptance of such technique in choir-singing. This alteration, however, resulted in a more expressive performance. The authors explain this by covert competition between sexually mature males for female attention, which inadvertently maximizes choir's collective output.

We hope that this volume will bring us closer to answer Darwin's quest (Darwin, 1890) to disclose the mystery of music's origin.

## Author Contributions

AN wrote the first draft of the manuscript. LP provided critical revision of the manuscript and important intellectual contributions. All authors contributed to the article and approved the submitted version.

## Conflict of Interest

The authors declare that the research was conducted in the absence of any commercial or financial relationships that could be construed as a potential conflict of interest.

## Footnotes

^1^This includes not only those papers that were submitted and published as part of our Research Topic, but also the ones that were not published (for various reasons).

^2^This refers not only to the matters of music theory and the performance practices of Western forms of music, but to the undocumented implicit music theories transmitted orally by music-makers within non-Western traditional music cultures. Confused by scientific terminology, they also often see scientific approach as essentially foreign to their music practice—despite their appreciation of scholarly attention to their respective traditions.

^3^Perhaps, a good demonstration of the unity of scientific and humanitarian approaches to music, common in the Soviet and modern Russian academia, is the partnership between both authors of this article: A. Nikolsky started his career as a professional composer, music theorist, and pianist, whereas L. Perlovsky—as a nuclear physicist. Somehow both authors met in their research on the origins and evolution of music.

^4^Nikolai Lobachevsky outlined the principles of the ideal scholarship in his speech “On the most important subjects of education” (1828), which he delivered upon his election as the rector of the Kazan University.

^5^Losev's semiotic studies eventually became most influential in the Soviet academia—laying out an encyclopedic Aristotelean-like theoretic foundation for all the disciplines in the empirical sciences and the arts. Losev's books “Music as a Subject of Logic” (1927) and “Dialectics of Artistic Form” (1927) reflected his lectures in Moscow Tchaikovsky Conservatory, where he taught aesthetics, and synthesized the neo-Platonic concept of music as a sounding number with Romantic ideas of symbolism in coining rational definitions of the notions of rhythm, melody, and harmony.

^6^Maykapar (1900) was a renowned composer, an outstanding concert pianist, and a distinguished pedagogue. He pioneered Helmholtz' approach in Russia to validate and reform the traditional way of teaching and making music—which included the psychoacoustic investigation of attention, melodic intonation, rhythm, timbre, tonality, and modality (Maykapar, 1900).

^7^Alexander Samoilov, assistant of Ivan Pavlov, and one of the developers of electrocardiography, was a virtuoso pianist and a musicologist who authored a number of publications on music theory (i.e., “Natural numbers in music,” “Musical ethnographic museum instruments and their musical tuning,” “Musical notation and its history”). He founded the Scientific-musicological Circle at Moscow University in 1902, which was later transferred to the Moscow Conservatory, where Sergei Taneyev and Samuel Maykapar subsequently took over his directorship. After moving to Kazan, Samoilov taught courses in math, physics, acoustics, music theory, and music history, in Kazan University.

^8^Second order beats are perceptible when two pure tones slightly deviate from simple, small-integer ratio relationships, e.g., 2:1 (octave), 3:2 (perfect 5th), and 4:3 (perfect 4th). Deviation from simple mathematical relationships results in some degree of chaos, perceived in dissonant intervals, e.g., tritones, minor 7ths.
